# Influence of the internet celebrity’ attributes of the host on the loyalty of users on live platforms

**DOI:** 10.1371/journal.pone.0310308

**Published:** 2024-09-27

**Authors:** Tingyu Cui, Panqian Dai, Jing Xu, Yixuan Lu, Wenxuan Wang

**Affiliations:** 1 Jiangsu Modern Logistics Research Base of Business School, Yangzhou University, Yangzhou, China; 2 School of Political Science and Public Administration, Suzhou University, Soochow University, Suzhou, China; Public Library of Science, UNITED KINGDOM OF GREAT BRITAIN AND NORTHERN IRELAND

## Abstract

Since 2019, live e-commerce has experienced significant growth, effectively driving consumption, with various e-commerce platforms, video platforms, enterprises, and businesses venturing into the live e-commerce sector. However, this development has brought forth several issues, one of which prominently pertains to the lack of customer loyalty. Hosts play a crucial role in live e-commerce. To investigate the impact of internet celebrity attributes on platform user loyalty, this study employed a survey questionnaire distributed on internet websites, collecting 200 valid samples. A research model based on the ABC attitude model was constructed, with customer satisfaction and customer trust as mediating variables. The findings indicate that the internet celebrity attributes of network popularity and interactivity positively influence customer loyalty through the mediation of customer satisfaction and customer trust. On the other hand, the host’s purpose as an internet celebrity attribute does not directly or indirectly affect customer loyalty. The primary reason for this discrepancy is that the host failed to establish trust and employ suitable methods to attain the goal of selling and promoting products. Consequently, platforms and businesses can enhance the network popularity of hosts through communication media, while fostering their literacy and professional skills. Moreover, strengthening communication and interaction between hosts and customers serves as a foundation for nurturing enduring and positive relationships.

## 1. Introduction

2016 was the first year of webcast, and various live platforms began to rise. After several years of development, webcast has become an indispensable part of the internet ecology, and live platforms that can communicate directly with audiences have attracted a large number of viewers [1, 2]. According to “The 45th China statistical report on Internet development”, in 2019, the total amount of product trade transactions generated by Mushroom Street through live e-commerce mode increased by nearly 140% year-on-year. In addition, the number of people who are keen on using live broadcast related services also increased by about 45%. However, in the face of the trend, the paradigm of live streaming content tends to be homogenized, consumer attention transfer costs are low, and traffic competition is becoming increasingly fierce. How to improve customer loyalty has become the top priority for the development of live streaming e-commerce platforms.

Compared with traditional e-commerce sales and communication methods, live e-commerce takes the host as the core, providing customers with more detailed product information, more timely and sufficient communication, highly personalized services, etc. through the host’s online live broadcast room display, recommendation, and real-time interaction with customers [3], thereby eliminating the uncertainty of customer online shopping and enhancing a good experience [4–6]. Therefore, hosts play a crucial role in cultivating customer stickiness in live streaming e-commerce. Some internet celebrity hosts with their own distinctive characteristics have attracted a large number of consumers to become loyal followers, creating numerous sales miracles. Such as Li Jiaqi has 79 million Taobao fans, with over 200 million views on the Double 11 live broadcast and sales reaching tens of billions of yuan. However, some scholars have found through empirical research that the familiarity of consumers with internet celebrity hosts does not have a significant impact on their loyalty [7]. Moreover, the overall evaluation of hosts by consumers is not high at present, and only a small proportion of internet celebrity hosts can be recognized. In view of this, this paper intends to explore from the perspective of hosts, which internet celebrity attributes of hosts can enable them to have high customer loyalty in the context of livestreaming economy? What path does the host’s internet celebrity attribute affect customer loyalty through? Is there a mediating effect between customer satisfaction and customer trust? etc. in order to provide theoretical support for live platforms to effectively utilize the characteristics of hosts, strengthen their own quality construction, and improve consumer retention.

Due to the relatively short period of time since the rise of live streaming e-commerce, academic research on it is still in its infancy. Most of the existing studies mainly describe the motivation of live streaming users in terms of entertainment, knowledge or experience sharing goals [8, 9], as well as gift-giving behaviors [10, 11]. Only a few scholars have conducted some research on the motivation of consumer buying behavior in e-commerce live platforms. For example, Jia utilized the SOR model, combined with social existence related theory and social facilitation theory, to study the influencing factors of consumers’ purchase and integration willingness in e-commerce platforms [12]. Qi used SOR theory as the basis of the theoretical model, and collected questionnaire data through the simulation experiment method to analyze the impact of the characteristics of the live streaming bandwagon platform on the consumer’s willingness to purchase [13]. These studies are mostly based on the SOR theory of unidirectional information dissemination as the analytical framework, while Netflix live streaming with goods, as a highly interactive, multi-dimensional and real-time interactive marketing mode with consumers, is not fully applicable to this theory. Some scholars have already paid attention to the great efficacy of internet celebrity hosts in the field of live e-commerce and explored which personalized characteristics of the hosts can promote consumer purchases. For example, from the perspective of information source characteristics, Meng classified the attributes of hosts into credibility, professionalism, skill, interactivity and attractiveness, and verified the impact on consumers’ purchase intention respectively [14]. Huang, on the other hand, launched a study on the impact of personal attributes such as personal charm, interactivity and professionalism of the lead host on consumers’ purchase intention [15]. However, this part of the study only focuses on the single purchase behavior of consumers, and does not extend to the study of customer loyalty. In fact, customer loyalty is more important for live e-commerce as consumers’ long-term sustained purchasing behaviors and attitudes. Studies on the loyalty of Internet platform users again focus on social media, short video platforms and other scenarios [16, 17], and there are fewer studies on customer loyalty in the live streaming with goods scenarios of hosts. In summary, this paper focuses on the issue of the impact of hosts attributes on customer loyalty in live e-commerce scenarios, and combines the previous studies and the characteristics of live e-commerce to define internet celebrity’ attributes of the host as network popularity, purposiveness, and interactivity, and explores the impact of the three attributes on customer loyalty via customer satisfaction and customer trust, respectively.

The remainder of this paper is organized as follows. Section 2 provides a comprehensive literature review of our topic and presents the research hypotheses based on the literature review. Section 3 provides a description of the data sources, variable measurements, and the theoretical hypothesized model. Section 4 describes the empirical analysis process. Section 5 gives the results of the study and discusses them. Finally, section 6 summarizes the theoretical and practical contributions, limitations, and future research directions of this study.

## 2. Theoretical hypotheses

### 2.1 Internet celebrity’ attributes of the host

With the popularity and development of the internet celebrity economy, some scholars have used the value of emotional experience as a mediating variable to explore how the internet celebrity’ attributes of hosts affect the willingness of audience participation in creating products [18], and begin to transfer the research object from the internet celebrity economy to the internet celebrity itself. While discussing the hosts’ attributes of internet celebrity, this paper used the attribute scale developed by Zhang Hao et al. [19] for fashion internet celebrity for reference, and selected network popularity and purposiveness within the scale. And considering that live broadcast has real-time interactive functions, it increases the interactive attributes of hosts [20], and finally defines the host internet celebrity attributes as three aspects: network popularity, purpose and interactivity. Network popularity is manifested in the spontaneous formation of reference groups with similar values and aesthetics among the host’s audience, who vigorously pursue and purchase the products marketed by the host [14]. Live broadcast enables the audience to observe the appearance and personality of the host. Therefore, they may appreciate the host because of his/her appearance, attitude, charm and talent [21], and become his / her fans, the popularity of the host increases as a result. In contrast to traditional celebrities (movie stars, dignitaries, sports personalities, etc.), internet celebrities have emerged with the popularity of social networks and live broadcast [22], and they influence the purchase intention of live viewers [23]. Regarding purposefulness, Sun and Wang [24] argue that internet celebrities are made for profit, presenting and constructing a self-image that can be easily consumed by others through increased popularity. In addition, many researchers have emphasized the grassroots attribute of internet celebrities, whose " down-to-earth " is characterized by a high frequency of interaction with their fans [25]. Live broadcast has strong real-time dynamic two-way interaction, and various types of live services usually provide interactive features such as text chat rooms, thus facilitating communication and interaction between live participants [26] and better evoking consumers’ desire to participate [14]. This is a reflection of the interactive attribute of the internet celebrity attribute, where the host will leave an impression of being approachable to fans.

### 2.2 Customer satisfaction

Customer satisfaction is usually defined as the degree to which a customer is satisfied during a particular transaction or purchase. Oliver [27] explained customer satisfaction with a transaction as an evaluation of the degree to which a customer’s specific needs and expectations are met after purchasing and using a product or service. The emotional state of a customer after consumption, which can be positive or negative, depends on prior expectations, the transactional experience or the perception of displeasure encountered in the service [28]. Satisfaction is achieved when the customer’s experience meets expectations. It has been argued that superior e-service quality positively affects customer satisfaction [1]. Perceived value represents the customer’s assessment of the quality of the product (and service) sought relative to its price and is expected to positively influence their level of satisfaction.

### 2.3 Customer trust

Shankar [29] believed that trust is an emotional attachment to a trading partner with whom one identifies. “Trust” is defined as the general belief that the other party will act in an ethical and socially appropriate manner and will not act opportunistically [30, 31]. In the e-commerce environment, customer trust is generally defined as consumer recognition and reliance on a website or company, brand, etc. in a risky online environment [32, 33]. They are confident that the information they receive is true and that the product they receive will be as expected [3]. Many academic studies have shown that businesses should focus on developing customer trust. Chadwick [34] considered that trust should occur before a consumer establishes a transactional behavior with a merchant. Strader and Shaw [35] found that when the price difference is not significant, consumers are more likely to choose the channel they trust to purchase products, and trust has become one of the key factors determining consumer behavior. McAllister [36] divides trust into cognitive and emotional trust, and he argues that people make emotional investments in trusting relationships, and that this emotion can associate merchants and customers together.

### 2.4 Customer loyalty

Copeland first defined the concept of customer loyalty in the early 1920s. For some time afterwards, scholars have considered customer loyalty only at the behavioral level. For example, Newman and Bell consider repeat buying behavior by customers as a symptom of customer loyalty; Tucker, on the other hand, defines customer loyalty as customers choosing the same product or service several times and reaching a fixed value. Later, it was suggested that customer loyalty should not only be judged by behavior, but also by having a positive attitude. The importance of attitude was also highlighted by Oliver et al. Sirdeshmukh et al. [37] defined customer loyalty as a behavioral tendency of customers who are willing to maintain a transactional relationship with a product or service provider. The definition of customer loyalty in an e-commerce environment is essentially the same as in a traditional marketplace, the difference being that companies need to use the Internet to foster customer loyalty.

### 2.5 Network popularity, customer satisfaction and customer trust

Network popularity means that the host generates network popularity after media publicity, enjoys a certain degree of fame among netizens, or passes the tier test divided by the platform, is recommended to a prominent position on the home page, and becomes the first choice for users to experience live broadcast [19]. ABC Attitude Theory states that an individual’s perception of the attitude object (the individual’s thought ideas resulting from processing known information about the attitude object) will affect the individual’s emotion, which will trigger the individual to perform a certain behavior. Here, the customer’s perception of the host’s network popularity, i.e., the customer is familiar with the host and recognizes his or her popularity and influence, which will affect the customer’s affective attitude [20], and then affect customer satisfaction and trust. That is, the nature of network popularity is the charisma effect of celebrities, and in the field of live e-commerce, consumers will be more willing to accept the viewpoints of products from hosts with high exposure and good reputation, and these hosts are more likely to be trusted by consumers. Based on the above analysis, following hypotheses are formulated:

H1: Customers’ cognition of network popularity of hosts has a positive effect on customer satisfaction in internet celebrity live.
H2: Customers’ cognition of network popularity of hosts has a positive effect on customer trust in internet celebrity live.

### 2.6 Purposiveness, customer satisfaction and customer trust

Purposiveness refers to that the hosts, as part of an e-commerce marketing tool, needs to accomplish the task of driving sales as well as promoting the brand [38]. Customers’ perception of the host’s purposiveness refers to the extent to which customers perceive the product promoted by the host during the live broadcast. As hosts have some expertise in their respective fields, they can be seen as opinion leaders in a sense, and they are more likely to trigger users’ emotional trust when they personally choose, try, introduce and share products. Some products used or recommended by hosts also cause people to follow and buy them [39]. Based on the above analysis, following hypotheses are formulated:

H3: Customers’ cognition of purposiveness of hosts has a positive effect on customer satisfaction in internet celebrity live.
H4: Customers’ cognition of purposiveness of hosts has a positive effect on customer trust in internet celebrity live.

### 2.7 Interactivity, customer satisfaction and customer trust

Interactivity is one of the greatest characteristics of Internet social media, and this attribute has successfully made the Internet stand out from the many communication media that are widely used [40]. Kotler [41] defines interactivity as the communication behavior between consumers and businesses. In this paper, interactivity is defined as the act of interaction between hosts and consumers that can be perceived. The live platform have enabled a culture of engagement to an unprecedented degree, with viewers being allowed to interact with the hosts and other viewers to a large extent. According to the research of Xue and Phelps [42], consumers are more likely to be influenced in their attitudes when interacting with opinion leaders than when the information is posted directly on the website. Some scholars believe that interactivity is not only a property possessed by some social platforms, it can also be a perceptual variable, i.e., interactivity as perceived by users. Social interactions can enhance the degree of customer trust [43] and customer satisfaction [44]. Based on the above analysis, following hypotheses are formulated:

H5: Customers’ cognition of interactivity of hosts has a positive effect on customer satisfaction in internet celebrity live.
H6: Customers’ cognition of interactivity of hosts has a s positive effect on customer trust in internet celebrity live.

### 2.8 Customer satisfaction, customer trust, customer loyalty and mediating effect

A large amount of marketing literature at home and abroad has proved that Internet customer satisfaction is an important antecedent exogenous derivative of loyalty in virtual consumption situations [45]. For example, Zhang Shengliang’s study confirms that Internet customer satisfaction positively affects their attitudinal and behavioral loyalty, and ultimately affects the profitability level of e-commerce enterprises [46]. Based on previous studies, it can be inferred that consumers have a higher tendency to repeat patronage when they are satisfied with the live platform or the anchor during the process of shopping for goods and enjoying services on the live platform.

H7: Customer satisfaction has a positive effect on customer loyalty in internet celebrity live.

In real e-commerce live streaming, first of all, the web popularity of the anchor will also have an impact on customer satisfaction, which in turn affects customer loyalty. The reason behind this is that since customers are satisfied with their purchases due to the celebrity effect of the anchor, they will inevitably be interested in further increasing their recognition of that anchor, which in turn leads to watching the live broadcast again and continued purchases. Secondly, customer satisfaction due to the anchor’s professional recommendation will also lead customers to continue purchasing or recommending the anchor to others [47]. Finally, the pleasant shopping experience resulting from customers’ interaction with anchors or others in the live broadcast will also attract customers to continue to patronize, e.g., some scholars have proposed that interactivity can promote the formation of quasi-social relationships between consumers and Netflix and influence customers’ decisions on repeat purchase behavior through the indirect effect of satisfaction [4]. Based on the above analysis, the hypothesis is proposed:

H7a: Customer satisfaction plays a mediating role in the relationship between network popularity and customer loyalty in internet celebrity live.
H7b: Customer satisfaction plays a mediating role in the relationship between purposiveness and customer loyalty in internet celebrity live.
H7c: Customer satisfaction plays a mediating role in the relationship between interactivity and customer loyalty in internet celebrity live.

In many previous studies, customer satisfaction and customer trust are both influential factors of customer loyalty [48], and customer satisfaction also has an impact on customer trust and ultimately contributes positively to the implementation of customer lock-in strategies by enterprises to improve the quality of customer relationships. Zeng Hui further confirms the importance of Internet customer satisfaction in the B2C model in maintaining customers’ continued trust [49]. As a corollary, in e-commerce live broadcasting customers who are satisfied with the online consumption experience will gradually build trust in the anchor.

H8: Customer satisfaction has a positive effect on customer trust in internet celebrity live.

Generally speaking, customer trust can make customers feel positive emotions towards anchors, which in turn increases their willingness to revisit and purchase [50], i.e., influencing customer loyalty. The mechanism behind this influence is that the dependability and intimacy generated by customer trust will lead to inertia psychology, which will lead to anchor favoritism and increase live streaming loyalty. At the same time, because live e-commerce is an Internet virtual transaction situation, trust can effectively reduce the subjective perception of consumers on the risk of information asymmetry, reduce the cost of information search and transaction costs, etc., and ultimately play a positive role in promoting customer loyalty [51].

H9: Customer trust has a positive effect on customer loyalty in internet celebrity live.

According to consumer identity theory, behind the anchor’s internet popularity is the consumer’s sense of identification with the personal traits of the web celebrity anchor and his or her attitude, and the more likely he or she will believe that the anchor’s products and services can meet expectations and raise expectations of good outcomes, thus maintaining the relationship with the current anchor. That is, customer identification generated by the anchor’s web popularity significantly enhances customer loyalty. In addition, if the anchor has already established trust with the customer in the live webcast, the anchor’s means and purpose of recommending the goods will also be more easily accepted by the customer, which in turn positively affects customer loyalty. Finally, the interaction unique to e-commerce live broadcasting also has a positive effect on stimulating customer trust and enhancing customer loyalty, because the interaction will make consumers feel valued by the anchor, as well as leaving the impression that the anchor is comprehensive in its presentation and service, and does not disguise hidden information [14]. Based on the above analysis, the hypothesis is proposed:

H9a: Customer trust plays a mediating role in the relationship between network popularity and customer loyalty in internet celebrity live.
H9b: Customer trust plays a mediating role in the relationship between purposiveness and customer loyalty in internet celebrity live.
H9c: Customer trust plays a mediating role in the relationship between interactivity and customer loyalty in internet celebrity live.

## 3. Methods

### 3.1 Data collection

Considering that the topic of this research is customer loyalty of live broadcasting e-commerce platform, the survey object is defined as the people who watch live broadcasting on a daily basis and have consumption experience during the viewing process. There are two specific survey channels, one is the questionnaire star platform, which is a professional online survey company with online questionnaire design, data collection and other functions, and is widely used by academics. The second is the questionnaire distribution through social software such as WeChat and QQ. The questionnaire survey began in February 2023 and ended in May, and a total of 249 completed questionnaires were retrieved, and after excluding unqualified questionnaires such as omissions and obvious similarities, 200 valid questionnaires were obtained. Generally speaking, the ratio of respondents to the number of questions should be 5:1 or higher, and in this study, there were 21 questions and 200 valid questionnaires to meet the requirements. The specific sample information is shown in Table 1. In terms of gender, the number of men accounts for nearly 20% of the total, while the remaining 80% are women; In terms of the age of the survey respondents, the sample was generally concentrated in the age range of 18–30. The number of people in each quantitative segment is relatively balanced in the monthly income survey. Overall, the sample is well representative of the entire study population. This study has been reviewed by the Ethics Committee of Yangzhou University School of Medicine and obtained a written review form with approval number YXYLL-2013-156.

**Table 1:** Demographic statistics.

Item	Type	Frequency	Percentage
Gender	male	41	20.5%
	female	159	79.5%
Age	<18	5	2.5%
	18–25	109	54.5%
	26–30	53	26.5%
	31–40	23	11.5%
	41–50	6	3.0%
	51–60	4	2.0%
	>60	0	0%
Monthly income	<2000	36	18%
	2000–4000	56	28%
	4000–6000	60	30%
	6000–8000	34	17%
	>8000	14	7%

### 3.2 Measurement

In this paper, the questionnaire was designed using a 7-level Likert scale, with different values from 1 to 7 referring to a process from "totally disagree" to "totally agree". The questionnaire measured six constructs: network popularity, purposiveness, interactivity, customer satisfaction, customer trust and customer loyalty. All measures of constructs were applied after adaptation from the existing literature base and scales, as shown in Table 2.

**Table 2:** Measurement items.

Construct	Sources	Item	Revised item
Network popularity	Zhang et al. [19]	They have a high degree of Internet exposure.	The host has a high degree of Internet exposure.
		They have a high degree of network popularity.	The host has a high degree of network popularity.
		They have a high degree of network awareness.	The host has a high degree of network awareness.
Purposiveness	Zhang et al. [19]	They aim to sell goods.	The host aims to sell goods.
		They are advertising.	The host is advertising.
		They aim to promote their products.	The host aims to promote the products.
Interactivity	Yao [40]	The internet celebrity always responds positively to my questions or topics.	The host always responds positively to my questions or topics.
		I will respond positively to the topic initiated by the internet celebrity.	I will respond positively to the topic initiated by the host.
		The internet celebrity often communicates with the public about products online.	The host will often communicate with the public about products.
		The internet celebrity can always respond to my questions or topics quickly.	The host can always respond to my questions or topics quickly.
Customer satisfaction	Ye [52]	Shopping on the Internet can satisfy my shopping demand.	The host can meet my shopping needs.
		I am satisfied with the experience of online shopping.	I am satisfied with the experience of shopping in the host’s live room.
		I feel happy after spending.	I feel happy after spending money in the host’s live room.
		I think online shopping is sensible.	I think shopping in the host’s live room is sensible.
Customer trust	Yao [40]; Jia [12]	I trust this information about the product that the internet celebrity said.	I trust this information about the product that the host said.
		This internet celebrity blogger is trustworthy.	The host is trustworthy.
		I believe the host is capable of handling online transactions.	I believe the host is capable of handling online transactions.
Customer loyalty	Lin [53]	I have purchased Ray-Ban products for many times.	I have shopped in the host’s live room many times.
		I will continue to buy Ray-Ban glasses products in the future.	I will continue to shop in the host’s live room in the future.
		I agree with the Ray-Ban brand and would recommend it to my friends and family.	I agree with the host and would recommend it to my friends and family.
		I am very happy to actively recommend to people around me to follow Ray-Ban.	

### 3.3 Measurement model

**Fig 1:**
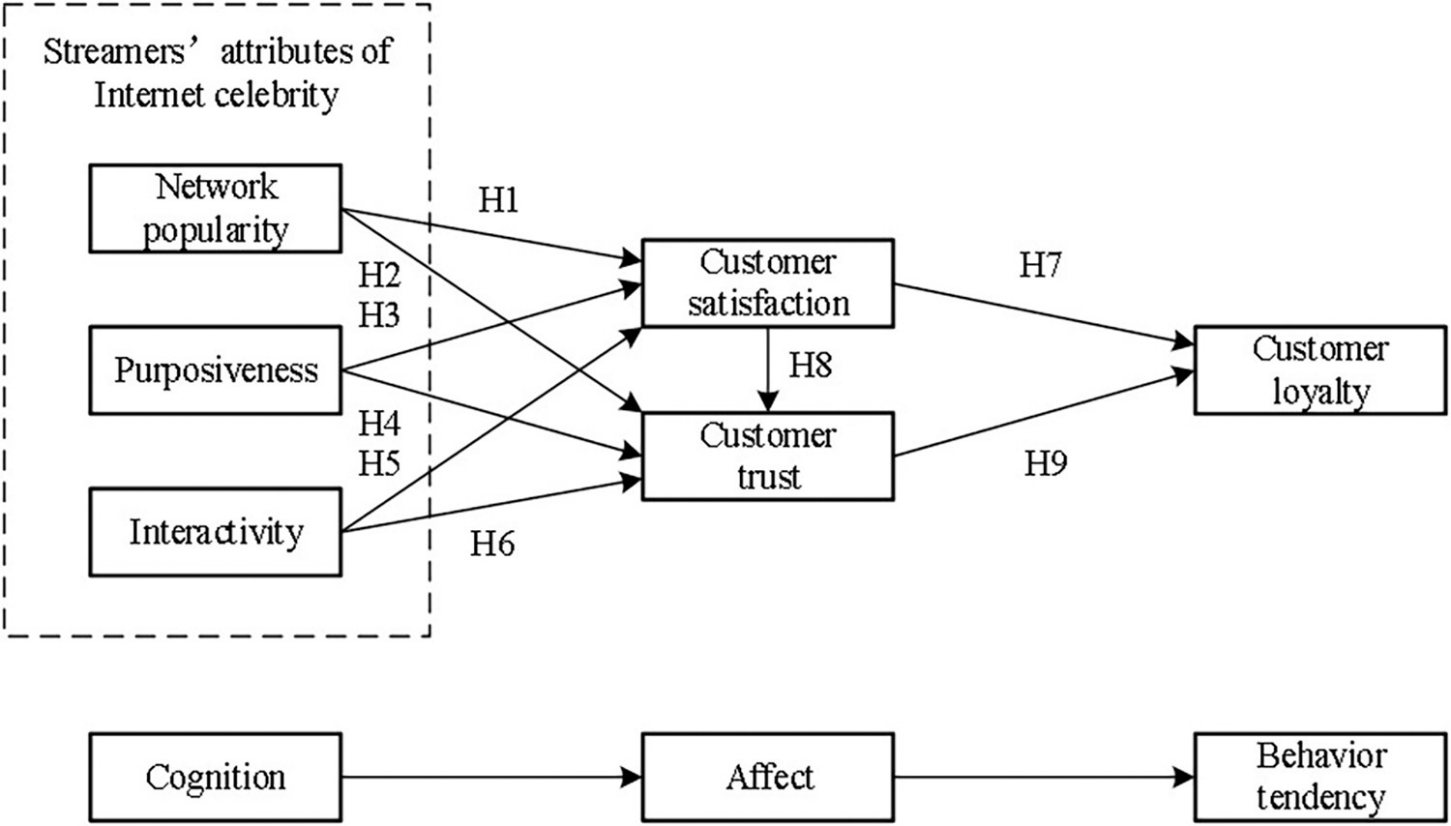
Research model.

In the 1960s, Hovland and Rosenberg created an attitude model constructed from three different elements: cognitive, affective, and behavioral response tendencies, named the ABC attitude model. In this model, cognition is the thinking thoughts that result from an individual’s processing of known information about an attitude object; Affect, on the other hand, refers to the more salient emotional state of an individual when confronted with an object; Behavioral response tendencies are the behaviors or behavioral tendencies that individuals do when confronted with an object. The ABC attitude model generalizes consumer attitudes in terms of the correlation between three components: cognitive, affective, and behavioral. This paper investigates the influence of host internet celebrity attributes on customer loyalty in the context of live e-commerce, based on the ABC attitude model, with host internet celebrity attributes as the perceived object of consumers; Satisfaction and trust are used as affective mediating variables to influence customer loyalty; customer loyalty is the performance of consumers in the behavioral stage, and the model diagram is shown in Fig 1.

## 4. Data analysis and results

### 4.1 Reliability test

The reliability test is mainly used to verify the internal consistency of the scale by calculating the Cronbach’s α coefficient of the scale. As can be seen from the data in Table 3, the Cronbach’s α values for all six dimensions involved in this questionnaire are greater than 0.8, which indicates that the high internal consistency of the scale. Meanwhile, the CR (composite reliability) values of all six dimensions in the validation analysis were greater than 0.7, indicating that each dimension has good composite reliability, i.e., all the measurement question items of each dimension consistently explain the dimension.

**Table 3:** Cronbach’s alpha, CR, and AVE value.

Construct	Cronbach’s α	CR	AVE	Number of items
Network popularity	0.824	0.974	0.925	3
Purposiveness	0.833	0.963	0.896	3
Interactivity	0.827	0.963	0.866	3
Customer satisfaction	0.823	0.974	0.904	3
Customer trust	0.810	0.971	0.919	3
Customer loyalty	0.846	0.963	0.896	3

### 4.2 Validity test

Validity examines the energy efficiency of each question item within the scale, i.e., it tests whether each question item is useful for the scale. This questionnaire was prepared to verify the validity of the scale by exploratory factor analysis. First, the relevant KMO and Bartlett’s tests were done for the questionnaire to assess whether the questionnaire could carry out exploratory factor analysis. According to the data in Table 4, the KMO measure of the scale is 0.908, which is greater than the standard value of 0.7. In addition, the approximate chi-square and degrees of freedom and p-values of Bartlett’s spherical test were 1998.861, 136, and 0.000, respectively, where the p-value was significantly lower than 0.01, thus completing a significance test with a significance level of 1% and the questionnaire was suitable for exploratory factor analysis. In addition, as shown in Table 3, the AVE (Average Variance Extracted) values for the six dimensions of the scale are greater than 0.5, indicating sufficient convergent validity.

**Table 4:** KMO and Bartlett’s test.

Kaiser-Meyer-Olkin measure of sampling adequacy		0.908
**Network popularity**	Approx. Chi-square	1998.861
	df	136
	Sig.	0.000

According to the data in Table 5, the six principal components were extracted using exploratory factor analysis with a cumulative contribution above 50%, thus indicating that the extracted factors have a good explanatory power for the variables.

**Table 5:** Total variance of interpretation.

Components	Initial eigenvalue			Extraction of squares loadings			Rotation of squares loadings		
	Total	Percentage of variance	Cumulative variance percentage	Total	Percentage of variance	Cumulative variance percentage	Total	Percentage of variance	Cumulative variance percentage
1	7.756	45.623	45.623	7.756	45.623	45.623	2.342	13.774	13.774
2	2.107	12.393	58.016	2.107	12.393	58.016	2.331	13.711	27.485
3	1.194	7.025	65.041	1.194	7.025	65.041	2.279	13.408	40.893
4	0.897	5.276	70.317	0.897	5.276	70.317	2.243	13.197	54.090
5	0.786	4.625	74.941	0.786	4.625	74.941	2.056	12.094	66.184
6	0.562	3.304	78.245	0.562	3.304	78.245	2.050	12.061	78.245

Based on the data in Table 6, question item Q6-2 has loadings higher than 0.5 on both dimensions at the same time, which cannot pass the validity test and is an invalid question item and should be deleted. The rest of the questions all had loadings higher than 0.5 on a single dimension only and were valid questions. According to the content of each question, the survey was divided into six dimensions: “network popularity”, “purposiveness”, “interactivity”, “customer satisfaction”, “customer trust” and “customer loyalty”.

**Table 6:** Rotating component matrix.

Construct	Item	Component					
		1	2	3	4	5	6
Customer loyalty	Q9-3	0.750					
	Q9-2	0.661					
	Q9-1	0.637					
Purposiveness	Q5-1		0.866				
	Q5-2		0.862				
	Q5-3		0.837				
Network popularity	Q3-1			0.802			
	Q3-2			0.776			
	Q3-3			0.754			
Interactivity	Q6-4				0.809		
	Q6-1				0.807		
	Q6-2	0.549			0.553		
Customer satisfaction	Q7-1					0.758	
	Q7-2					0.661	
	Q7-3					0.538	
Customer trust	Q8-1						0.786
	Q8-3						0.733

### 4.3 Correlation analysis

Correlation analysis is mainly used to assess the correlation of different variables with each other, which can be judged by Pearson coefficient in practice. If the p-value is greater than 0, then the variables are positively correlated with each other; if the p-value is less than 0, then the variables are negatively correlated with each other.

According to Table 7, the P-value of network popularity, purposiveness, interactivity, customer satisfaction and customer loyalty are all positive, indicating that there is a positive relationship between network popularity, purposiveness, interactivity, customer satisfaction and customer trust. In addition, the p-values of customer satisfaction, customer trust and customer loyalty are all greater than zero, and there is a positive relationship between them.

**Table 7:** Pearson correlation coefficient.

Construct	Network popularity	Purposiveness	Interactivity	Customer satisfaction	Customer trust	Customer loyalty
Network popularity	1					
Purposiveness	0.248**	1				
Interactivity	0.511**	0.146*	1			
Customer satisfaction	0.602**	0.243**	0.706**	1		
Customer trust	0.553**	0.260**	0.589**	0.675**	1	
Customer loyalty	0.533**	0.245**	0.668**	0.768**	0.674**	1

Note: P < 0.01**, P < 0.05*.

### 4.4 Regression analysis

Regression analysis is a method used to establish a functional relationship between an independent variable and a dependent variable, which can be used to characterize the degree and direction of the influence of the independent variable on the dependent variable. Because of the presence of mediating variables in the model of this paper, SPSSAU analysis software was used, and Bootstrap sampling method was used for testing.

#### 4.4.1 Network popularity and customer loyalty

According to the data in Table 8, the values of R^2^ are all greater than 0.4, indicating a good fit of the model.

**Table 8:** Mediating effect test.

	Customer satisfaction	Customer trust	Customer loyalty	Customer loyalty
Constants	0.662 (1.936)	1.083 (2.711**)	0.455 (1.103)	-0.148 (-0.417)
Purposiveness	0.083 (1.875)	0.115 (2.229*)	0.113 (2.110*)	0.042 (0.928)
Interactivity	0.470 (10.007**)	0.365 (6.651**)	0.517 (9.119**)	0.191 (3.195**)
Network popularity	0.325 (5.597**)	0.336 (4.953**)	0.275 (3.915**)	0.028 (0.428)
Customer satisfaction				0.499 (6.442**)
Customer trust				0.252 (3.808**)
Sample size	200	200	200	200
R^2^	0.585	0.447	0.507	0.655
Adjusted R^2^	0.578	0.438	0.499	0.646
F	F (3,196) = 91.920, p = 0.000	F (3,196) = 52.784, p = 0.000	F (3,196) = 67.087, p = 0.000	F (5,194) = 73.564, p = 0.000

Note: * p<0.05

** p<0.01.

Based on Table 9 it can be seen that: at the level of total effect, the total effect of network popularity on customer loyalty shows a significance at the level of 0.01 (t = 3.915, P = 0.000 < 0.01); In terms of direct effects, network popularity did not have a significant effect relationship on customer loyalty (t = 0.428, p = 0.669 > 0.05), which means that direct effects do not exist.

**Table 9:** Effect path analysis.

Effect	Effect path	Effect	SE	t	p	LLCI	ULCI
Direct effect	Network popularity ⇒ Customer loyalty	0.028	0.065	0.428	0.669	-0.100	0.155
Indirect effect	Network popularity ⇒ Customer satisfaction	0.325	0.058	5.597	0.000	0.211	0.439
	Network popularity ⇒ Customer trust	0.336	0.068	4.953	0.000	0.203	0.469
	Customer satisfaction ⇒ Customer loyalty	0.499	0.077	6.442	0.000	0.347	0.650
	Customer trust ⇒ Customer loyalty	0.252	0.066	3.808	0.000	0.122	0.382
Total effect	Network popularity ⇒ Customer loyalty	0.275	0.070	3.915	0.000	0.137	0.412

Note: LLCI refers to the lower limit of the 95% interval of the estimate, ULCI refers to the upper limit of the 95% interval of the estimate.

The indirect effects are divided into two, which are network popularity through customer satisfaction and customer trust to influence customer loyalty again.

According to the data in Table 10, the regression coefficient value of network popularity ⇒customer satisfaction ⇒customer loyalty was 0.162 (= 0.325*0.499); The regression coefficient value of network popularity ⇒customer trust⇒ customer loyalty was 0.085 (= 0.336*0.252).

**Table 10:** Effect path analysis.

Effect path	Effect	Boot SE	BootLLCI	BootULCI	z	p
Network popularity ⇒ Customer satisfaction ⇒ Customer loyalty	0.162	0.042	0.091	0.255	3.832	0.000
Network popularity ⇒ Customer trust ⇒ Customer loyalty	0.085	0.034	0.022	0.156	2.473	0.013

Note: BootLLCI refers to the lower limit of the 95% interval for Bootstrap sampling, BootULCI refers to the upper limit of the 95% interval for Bootstrap sampling.

For the test of mediating effect of customer satisfaction when the effect of network popularity on customer loyalty, the 95% confidence interval of the a*b test coefficient value does not include the number 0 (95% CI:0.091 to 0.255). Thus, it shows that customer satisfaction has a mediating role when it comes to the effect of network popularity on customer loyalty. Network popularity will first have an impact on customer satisfaction, and then through customer satisfaction then to influence customer loyalty.

For the test of mediating effect of customer trust when the effect of network popularity on customer loyalty, the 95% confidence interval of the a*b test coefficient value does not include the number 0 (95% CI:0.022 to 0.156). Therefore, it shows that customer trust plays an intermediary role in the influence of network popularity on customer loyalty. Network popularity first affects customer trust, which in turn affects customer loyalty through the effect of customer trust.

As shown in Table 11, the mediating effect of customer satisfaction accounted for 58.91% and the mediating effect of customer trust accounted for 30.91% when the influence of network popularity on customer loyalty.

**Table 11:** Proportion of effect.

Effect	Effect path	Regression estimated value	Proportion of effect
Direct effect	Network popularity ⇒ Customer loyalty	0.028	10.18%
Indirect effect	Network popularity ⇒ Customer satisfaction ⇒ Customer loyalty	0.162	58.91%
	Network popularity ⇒ Customer trust ⇒ Customer loyalty	0.085	30.91%
Total effect	Network popularity ⇒ Customer loyalty	0.275	100%

#### 4.4.2 Network popularity and customer loyalty

Table 12 shows the effect path analysis. In terms of total effect, the total effect of purposiveness on customer loyalty showed a significant level of 0.05(t = 2.110, p = 0.036<0.05); In terms of direct effects, purposiveness did not have a significant effect relationship on customer loyalty (t = 0.928, p = 0.355 > 0.05), which means that direct effects do not exist.

**Table 12:** Effect path analysis.

Effect	Effect path	Effect	SE	t	p	LLCI	ULCI
Direct effect	Purposiveness ⇒ Customer loyalty	0.042	0.046	0.928	0.355	-0.047	0.132
Indirect effect	Purposiveness ⇒ Customer satisfaction	0.083	0.044	1.875	0.062	-0.004	0.170
	Purposiveness ⇒ Customer trust	0.115	0.052	2.229	0.027	0.014	0.217
	Customer satisfaction ⇒ Customer loyalty	0.499	0.077	6.442	0.000	0.347	0.650
	Customer trust ⇒ Customer loyalty	0.252	0.066	3.808	0.000	0.122	0.382
Total effect	Purposiveness ⇒ Customer loyalty	0.113	0.054	2.110	0.036	0.008	0.218

Note: LLCI refers to the lower limit of the 95% interval of the estimate, ULCI refers to the upper limit of the 95% interval of the estimate.

Indirect effects are divided into two, which are purposiveness to influence customer loyalty through customer satisfaction and customer trust again.

According to the data in Table 13, the 95% interval of a*b test coefficient values includes the number 0 (95% CI: -0.004 to 0.089) for the test of mediating effect of customer satisfaction when the effect of purposiveness on customer loyalty. Thus, customer satisfaction does not have a mediating role when illustrating the effect of purposiveness on customer loyalty. The 95% interval of a*b test coefficient values includes the number 0 (95% CI: -0.004 to 0.081) for the test of mediating effect of customer trust when purposiveness affects customer loyalty, thus indicating that there is no mediating effect of customer trust when purposiveness affects customer loyalty.

**Table 13:** Indirect effect analysis.

Effect path	Effect	Boot SE	BootLLCI	BootULCI	z	p
Purposiveness ⇒ Customer satisfaction ⇒ Customer loyalty	0.041	0.023	-0.004	0.089	1.770	0.077
Purposiveness ⇒ Customer trust ⇒ Customer loyalty	0.029	0.022	-0.004	0.081	1.335	0.182

Note: BootLLCI refers to the lower limit of the 95% interval for Bootstrap sampling, BootULCI refers to the upper limit of the 95% interval for Bootstrap sampling.

#### 4.4.3 Interactivity and customer loyalty

Based on the Table 14 below, it can be observed that at the level of total effect, the total effect of interactivity on customer loyalty shows a significance at the level of 0.01 (t = 9.119, p = 0.000 < 0.01). In terms of direct effect, interactivity also has a significant effect relationship on customer loyalty (t = 3.195, p = 0.002 < 0.01), which means that there is a direct effect.

**Table 14:** Effect path analysis.

Effect	Effect path	Effect	SE	t	p	LLCI	ULCI
Direct effect	Interactivity ⇒ Customer loyalty	0.191	0.060	3.195	0.002	0.074	0.308
Indirect effect	Interactivity ⇒ Customer satisfaction	0.470	0.047	10.007	0.000	0.378	0.562
	Interactivity ⇒ Customer trust t	0.365	0.055	6.651	0.000	0.257	0.472
	Customer satisfaction ⇒ Customer loyalty	0.499	0.077	6.442	0.000	0.347	0.650
	Customer trust ⇒ Customer loyalty	0.252	0.066	3.808	0.000	0.122	0.382
Total effect	Interactivity ⇒ Customer loyalty	0.517	0.057	9.119	0.000	0.406	0.628

Note: LLCI refers to the lower limit of the 95% interval of the estimate, ULCI refers to the upper limit of the 95% interval of the estimate.

Indirect effects could be divided into two parts: interactivity affected customer loyalty through customer satisfaction and interactivity affected customer loyalty through customer trust.

In the Table above, interactivity ⇒customer satisfaction ⇒customer loyalty, the regression coefficient value of this model is 0.234 (= 0.470*0.499); And the value of regression coefficient of interactivity ⇒ customer trust ⇒ customer loyalty influence model is 0.092 (= 0.365*0.252).

According to the data in Table 15, the 95% interval of a*b test coefficient values does not include the number 0 (95% CI: 0.144 to 0.348) for the test of mediating effect of customer satisfaction when interactivity has an impact on customer loyalty. Thus, customer satisfaction has a mediating role when illustrating the effect of interactivity on customer loyalty. Interactivity first has an impact on customer satisfaction, and then on customer loyalty through customer satisfaction.

**Table 15:** Indirect effect analysis.

Effect path	Effect	Boot SE	BootLLCI	BootULCI	z	p
Interactivity ⇒ Customer satisfaction ⇒ Customer loyalty	0.234	0.052	0.144	0.348	4.480	0.000
Interactivity ⇒ Customer trust ⇒ Customer loyalty	0.092	0.035	0.027	0.167	2.611	0.009

Note: BootLLCI refers to the lower limit of the 95% interval for Bootstrap sampling, BootULCI refers to the upper limit of the 95% interval for Bootstrap sampling.

For the test of the mediating role of customer trust when interactivity has an impact on customer loyalty, the 95% interval of a*b test coefficient values does not include numbers 0 (95% CI: 0.027~0.167). Thus, customer trust has a mediating role when illustrating the impact of interactivity on customer loyalty.

As illustrated in Table 16 below, the direct effect of interactivity on customer loyalty accounted for 36.94%; the mediating effect of customer satisfaction accounted for 45.26%; and the mediating effect of customer trust accounted for 17.79%.

**Table 16:** Proportion of effect.

Effect	Effect path	Regression estimated value	Proportion of effect
Direct effect	Interactivity ⇒ Customer loyalty	0.191	36.94%
Indirect effect	Interactivity ⇒ Customer satisfaction ⇒ Customer loyalty	0.234	45.26%
	Interactivity ⇒ Customer trust ⇒ Customer loyalty	0.092	17.79%
Total effect	Interactivity ⇒ Customer loyalty	0.517	100%

## 5. Discussion

Based on the ABC attitude model, based on the logical structure that cognition influences emotion, and emotion then influences behavior, this study divides the internet celebrity’ attributes of the host into three dimensions, namely, network popularity, purposiveness, and interactivity, and studies the influence of host Netflix attributes on customer loyalty with customer satisfaction and customer trust as mediating variables.

1. This research found that the direct effect of the host’s internet popularity on customer loyalty is not significant, but internet popularity has a significant positive effect on both customer satisfaction and customer trust, and customer satisfaction and customer trust in turn have a mediating role in the relationship between internet popularity and customer loyalty. That is, Internet popularity first affects customer satisfaction and customer trust, and then acts on customer loyalty through two mediating variables, in which the mediating effect of customer satisfaction accounts for a higher proportion than customer trust. This finding was able to be proved in the same way in a number of studies addressing the purchase intentions of live e-commerce users. For example, E. Djafarova explored the impact of web celebrity Instagram profiles on young female users’ purchase decisions and found that endorsements by online celebrities were considered credible [22]. Wang pointed out that host network popularity significantly and positively affects users’ emotional experience value, while emotional experience value has a significant positive effect on users’ willingness to participate in value co-creation [18]. That is, the higher the host’s network popularity represents the more the host is recognized by the live customers, and when the customers have similar preferences and values with the host, the host’s product recommendations will be more easily accepted and trusted, and the accumulation of long-lasting customer satisfaction and customer trust will generate customer stickiness.
2. The results of the study indicate that there is no direct effect of hosts’ purpose on customer loyalty; purpose has a non-significant effect on customer satisfaction, but a significant positive effect on customer trust; and neither customer satisfaction nor customer trust has a significant mediating role in the relationship between purpose and customer loyalty. This is an unexpected finding, in the past, it is usually believed that stars and celebrities actively recommend products through advertisements, etc., and show the purpose of sales can stimulate consumers to buy [54]. However, the scenario studied in this paper is the selling of goods by Netflix hosts in live e-commerce, and the form of live broadcasting will amplify the words and actions of the host and interact with consumers in real time, so if the host does not take the appropriate way to achieve the purpose of promoting the product, it will be regarded by consumers as hype and self-marketing, and will not be able to stimulate customer satisfaction and customer followers.

This research found that host interactivity has a significant direct effect on customer loyalty; interactivity has a significant positive effect on customer satisfaction as well as customer trust; and customer satisfaction and customer trust also have a significant mediating effect in the relationship between interactivity and customer loyalty, with customer satisfaction accounting for a higher proportion of the mediating effect. In this regard, previous studies have similar findings to this study, but their studies have different mediating variables and influence paths than this study. For example, Meng, based on psychological arousal theory, verified that host interactivity can arouse consumers’ purchase intention through mediating variables such as social presence and identification [14]. Yao pointed out that the perceived interactivity of e-commerce Netflix has a positive contribution to the formation of a quasi-social relationship between consumers and Netflix, which indirectly acts on consumers’ repeat purchase intention through satisfaction [40].

## 6. Conclusions

This research has the following three theoretical contributions: first, it improves the research on customer loyalty in live e-commerce platforms and explores the key influencing factor of the host’s web popularity attribute. Second, combining the characteristics of live e-commerce, the host’s netizen attributes are defined as network popularity, purposiveness and interactivity, and through the study, it is found that the host’s network popularity and interactivity have an impact on customer loyalty, but the purposiveness does not have any impact. Third, using the ABC attitude model, customer satisfaction and customer trust are selected as mediating variables to verify the relationship path of consumers watching live webcam and forming customer loyalty.

Our findings also have some practical contributions for live e-commerce platforms and hosts. This paper demonstrates that the Netflix attribute of hosts has a positive impact on the customer loyalty of e-commerce platforms, i.e., the cultivation of excellent hosts is an important way to retain consumers. On the one hand, it can draw the attention of e-commerce platforms to the cultivation of hosts and the development of targeted marketing programs, and on the other hand, it can help hosts carry out a clear self-knowledge and precise positioning, and grow up to be Netflix hosts. Specifically, first, this paper finds that host network popularity has a positive impact on customer loyalty through customer satisfaction and customer trust. Accordingly, live e-commerce platforms should increase the publicity and promotion of the platform’s high-popularity hosts, or directly invite celebrities and stars favored by potential customers to serve as hosts. At the same time, the host should also take the initiative to strengthen their own characteristics mining and professional knowledge, through the provision of higher quality live with goods service to enhance customer satisfaction and trust. Second, host purposiveness has no significant effect on customer satisfaction and customer loyalty. Accordingly, hosts should pay attention to the ways and means of selling and promoting products, and be prudent in marketing to avoid triggering customer resentment. Third, host interactivity has a direct effect on customer loyalty and a mediated effect through customer satisfaction and customer trust. Accordingly, the host can improve the frequency of interaction and communication with customers, fully answer customer questions about the product; at the same time, focus on the diversity of interactive forms, such as random and customers with the microphone, arranging a number of lucky draws, gifts and other activities to strengthen the sense of participation of customers.

The limitations and shortcomings of this paper are mainly two: First, the model needs to be further improved and studied. In this study, from the host perspective, the host internet celebrity attributes are selected as the variables in the perception stage, and the model can be enriched by adding variables such as perceived value and perceived risk in the future by considering the influence of product factors. Second, the effective sample data size is small. Because of the epidemic, all questionnaires were collected online, and a total of 200 valid questionnaires were returned. The overall data volume is small, and the sample size can be further expanded in the future to make the study findings more convincing.

## Supporting information

S1 Data Original empirical study data. (XLSX)
